# Diagnostic evaluation of food-related allergic diseases

**DOI:** 10.1186/1710-1492-5-2

**Published:** 2009-10-22

**Authors:** John Eckman, Sarbjit S Saini, Robert G Hamilton

**Affiliations:** 1 Division of Allergy and Clinical Immunology, Department of Medicine, Johns Hopkins University School of Medicine, Baltimore, Maryland, USA; 2 Department of Pathology, Johns Hopkins University School of Medicine, Baltimore, Maryland, USA

## Abstract

Food allergy is a serious and potentially life-threatening problem for an estimated 6% of children and 3.7% of adults. This review examines the diagnostic process that begins with a patient's history and physical examination. If the suspicion of IgE-mediated food allergy is compelling based on the history, skin and serology tests are routinely performed to provide confirmation for the presence of food-specific IgE antibody. In selected cases, a provocation challenge may be required as a definitive or gold standard reference test for confirmation of IgE mediated reactions to food. Variables that influence the accuracy of each of the diagnostic algorithm phases are discussed. The clinical significance of food allergen-specific IgE antibody cross-reactivity and IgE antibody epitope mapping of food allergens is overviewed. The advantages and limitations of the various diagnostic procedures are examined with an emphasis on future trends in technology and reagents.

## Introduction

Approximately 6% of children and 3.7% of adults experience IgE-mediated allergic symptoms following the ingestion of food [1]. This contrasts with approximately 20% of the population that alters their diet for a perceived adverse reaction to food [2]. The allergist has the challenge of accurately identifying immunologically and non-immunologically-mediated reactions in the setting of this perception using information provided by the patient's history, skin and serology testing for food-specific IgE and food challenges.

A number of general issues must be considered when reviewing studies on the diagnosis of food allergy. These considerations include the characteristics of the patient population in individual studies, the instrumentation and interpretation of allergen-specific IgE skin and serology testing and variations in food challenge protocols [3].

This review examines the diagnostic process that begins with a patient's history and physical examination. We will overview considerations involved in skin testing and then focus on specific IgE testing, which has become of paramount importance in both diagnosing and following the natural history of food allergy. We highlight potential problems with the "gold standard" of food allergy diagnosis, the double-blinded, placebo-controlled food challenge. We then review the importance of considering cross-reactivity in the interpretation of skin testing and specific-IgE testing while discussing new technologies that may help decipher the degree of cross-reactivity. Finally, we mention the experimental studies of food-allergen epitope mapping in predicting the natural history of milk and egg allergy.

### Clinical history

The patient's history and physical examination are the foundation for the diagnosis of food allergy. The first goal is to distinguish whether the patient's reaction has an immunologic or a non-immunologic basis. Immunologic reactions include immediate-type, IgE-mediated reactions that involve the skin (pruritus, urticaria, angioedema, flushing), GI tract (oral pruritus, nausea, vomiting, diarrhea), nasal/respiratory tract (nasal congestion, rhinnorhea, ocular pruritus, sneezing, nasal pruritus, laryngeal edema, wheezing, shortness of breath) and/or the cardiovascular system (light-headedness, syncope, hypotension). These reactions can lead to death [4,5]. These symptoms typically begin within an hour of ingestion of the culprit food. The foods most commonly involved in food allergy are cow's milk, hen's egg, peanuts, tree nuts, seeds, soy, wheat, fish and crustaceans [6]. "Oral allergy syndrome" is an IgE-mediated reaction to fresh fruit, and less frequently nuts and vegetables, due to cross-reactivity to aeroallergens such as birch tree pollen or ragweed that cause oral pruritus, tingling and/or angioedema of the lips, palate, tongue or oropharynx [7].

Other food-mediated immunological or non-immunological reactions have different history and physical examination features from immediate-type hypersensitivity reactions. Conditions with both non-IgE and IgE based mechanisms include eosinophilic gastrointestinal disorders and atopic dermatitis. Types of cell-mediated food hypersensitivity include food-induced enterocolitis, food-induced pulmonary hemosiderosis (Heiner's syndrone), celiac disease, contact dermatitis and dermatitis herpetiformis. Non-immunologic reactions include lactose intolerance or other problems with food digestion. This review will focus on the diagnosis of immediate-type, IgE-mediated food allergy.

### Skin prick testing

In conjunction with the history and physical exam, diagnostic skin testing is a cornerstone in the evaluation of food allergy. It offers an in-office, rapid, and sensitive assessment of allergen sensitization.

General considerations of skin testing should be discussed first before exploring the specific details of food allergen skin testing. Extensive variability exists in skin prick test devices, skin testing techniques used, and the grading and interpretation of results [8-10]. Each variable needs to be carefully considered before extrapolating the conclusions from a published study to one's own clinical practice [10]. Inter-physician variation in scoring and interpretation of skin tests is of particular concern in tests that are not strongly positive or definitively negative [8].

Extending this discussion to food allergy, none of the food extracts used in diagnostic skin testing have been standardized, and therefore, significant heterogeneity in allergenic protein content and variability in the ultimate biological potency of these extracts often occurs between lots. Fruits and vegetables produce extracts that contain particularly labile allergens, and thus the use of fresh produce may offer increased sensitivity using the prick-prick method [11]. Intradermal skin testing can also be associated with systemic reactions and it is generally not recommended for the diagnosis of food allergy [12]. In one study, no patient with a positive intradermal skin test and a negative SPT to food had a positive double-blind placebo controlled food challenge (DBPCFC) [12].

Age must also be taken into account when assessing skin test reactivity. Children younger than 2 years of age may have less skin reactivity and thus smaller wheals than older children. Children less than 1 year of age may have IgE-mediated allergic disease related to a particular food in the absence of skin test reactivity [13]

While the diagnostic sensitivity of negative puncture skin test results is >95% in ruling out food allergy [2], its diagnostic specificity is limited. A larger puncture skin test wheal size in conjunction with a positive clinical history has been correlated with an increased likelihood of a positive open food challenge [14-17]. Specifically, using a lancet puncture technique and an open food challenge for confirmation, Sporik et al. demonstrated no negative challenges if puncture skin test wheal sizes were ≥ 8 mm for cow's milk or peanut and ≥ 7 mm for hen's egg [16]. Other studies have reported similar findings specifically for hen's egg [18] and tree nut allergy [19].

### Specific IgE testing

To date, the ImmunoCAP (Phadia, Uppsala, Sweden) has been the only clinically used IgE antibody immunoassay that has been systematically evaluated for its predictive value in food allergy studies. Recent studies have shown that the Immulite (Siemens Healthcare Diagnostics, Los Angeles, CA, USA) may overestimate specific IgE measurements in comparison to ImmunoCAP results [20,21]. Moreover, the Turbo RAST (currently HYTECH-288, Hycor Biomedical-Agilent, Garden Grove, CA, USA) reportedly overestimated egg-specific IgE but underestimated IgE antibody levels to birch and *D. farinae *in another study in comparison to the ImmunoCAP [20]. These data emphasize that the different clinically-used IgE antibody autoanalyzers detect different populations of IgE antibody.

While the majority of research performed to date [1,2,21-26] on the predictive power of quantitative food-specific IgE antibody levels has been performed using the ImmunoCAP System, it is likely that this is not the only assay method that possesses the ability to predict individuals who will experience positive food challenges. The research to investigate the predictive power of other specific IgE assays has simply not yet been performed. A 2008 Clinical Laboratory Standards Institute consensus guideline on quantitative IgE antibody methods [27] emphasizes that each of the principal serological IgE antibody assays used in clinical laboratories worldwide measures a different population of IgE antibody for any given allergen specificity. Thus, IgE antibody results generated with one method should thus not be used to make predictive clinical judgments with data in the literature generated using another assay method [20,27]. The different quantitative IgE antibody results among assays is most likely not the result of an inherent assay design issue or their total IgE calibration systems that are standardized to the same World Health Organization 75/502 IgE reference preparation. Rather, these different IgE antibody results are more likely to be a result of differential expression on allergenic molecules/epitopes on the allergen-containing reagent in each of these assays.

A new chip-based IgE antibody technology has emerged to enhance the food allergen-specific IgE antibody data that are available to both the clinician and the patient. The microarray chip technology [28,29] has been commercialized in the form of the ImmunoCAP-ISAC or Immuno Solid phase Allergen Chip (VBC Genomics-Vienna, Austria; Phadia, Uppsala, Sweden). It currently has 103 native/recombinant component allergens from 43 allergen sources that are dotted in triplicate onto glass slides. Twenty microliters of serum are pipetted onto the chip and antibodies specific for the allergens attached to the chip surface bind during a 2 hour incubation period. Following a buffer wash, bound IgE is detected with a fluorescently-labeled anti-IgE. The chip is read in a fluorometer and fluorescent signal units are interpolated into ISU or ISAC units as semi-quantitative estimates of specific IgE antibody in the original serum. The analytical sensitivity of the ISAC varies as a function of the particular allergen specificity and is generally viewed as less than the ImmunoCAP system when the same allergens are coupled to sponge allergosorbent. This device has been providing clinical data to clinicians in Europe for several years, but is not yet cleared by North American regulatory agencies for clinical use.

Historically, specific IgE testing has been considered by the allergist to be less sensitive than skin testing in the diagnosis of food allergy [30]. However, during the 21^st ^century, serological measurements of food-specific IgE antibody have become vital to the evaluation of food allergy, especially in children. Serological IgE antibody assays have the advantage of providing quantitative values that can aid in predicting with high certainty the presence of clinically significant food allergy, and thereby decreasing the need for food challenges. While this has been clearly demonstrated by Sampson and Ho [26] with the ImmunoCAP, future work needs to be done to evaluate the predictive cutpoints of the other IgE antibody assay methods.

Food-specific IgE measurements on retrospectively evaluated sera were used to develop 95% positive predictive values for food allergy to milk, egg, peanut and fish in a group of children with atopic dermatitis [26]. These cutoff values were then confirmed in a prospective study of a similar patient population to achieve 90% diagnostic specificity threshold values that can be used to avoid the need for food challenge [25]. Predictive values for walnut have also been developed [23]. Importantly, different predictive values have emerged beyond the initial studies which represent differences in diet, demographics (especially age), disease states (e.g. presence or absence of atopic dermatitis) of the study populations, and the challenge protocols (see Table 1). Therefore, it is critical to consider these factors when extrapolating the clinical relevance of the quantitative measures of IgE antibody. Specific IgE values have also been used to determine the appropriateness of a food challenge. For instance, a specific IgE level of 2 kUa/L in a group of children with a high prevalence of atopic dermatitis represented an approximately 50% likelihood of passing a food challenge to milk, egg or peanut [31]. This 50% likelihood is considered an acceptable risk/benefit level for a food challenge [31].

**Table 1:** Comparison of studies reviewing the positive predictive values of food specific IgE testing.

**Study**	**No. subjects**	**% Atopic Dermatitis**	**Average Age (years)**	**Study design**	**Food**	**Total IgE median kU/L(range)**	**PPV value %/Specific IgE level** **(kU/L)**	**Sens. for IgE level**	**Spec. for IgE level**
Sampson HA and Ho DG [26]	196	100%	5.2	Retrospective DBPCFC in 64%	Cow's milk	3000 (100-40,000)	95%/32	51%	98%

Sampson HA [25]	62	61%	3.8	Prospective DBPCFC in 34%	Cow's milk	*	95%/15	57%	94%

Garcia-Ara C et al[58]	170	23%	0.4	Prospective open controlled challenge in 95%	Cow'smilk	*	95%/5	30%	99%

Celik-Bilgili S et al[22]	398	88%	1.1	Prospective DBPCFC or open challenge in all	Cow'smilk	*	90%/88.8	*	*

Sampson HA and Ho DG[26]	196	100%	5.2	Retrospective DBPCFC in 64%	Hen's egg	3000 (100-40,000)	95%/6	72%	90%

Sampson HA [25]	75	61%	3.8	Prospective DBPCFC in 33%	Hen's egg	*	98%/7	61%	95%

Celik-Bilgili S et al. [22]	227	88%	1.1	Prospective DBPCFC or open challenge in all	Hen's egg	*	95%/12.6	*	*

Boyano Martinez T et al. [61]	81	43%	1.3	Prospective, Open controlled challenge in all	Egg white	40(3-597)	94%/0.35	91%	77%

Osterballe M et al[62]	56	100%	2.2	Prospective, Open challenge in all	Egg white	*	100%/1.5	60%	100%

Sampson HA and Ho DG [26]	196	100%	5.2	Retrospective DBPCFC in 64%	Peanut	3000 (100-40,000)	95%/15	73%	92%

Sampson HA[25]	68	61%	3.8	Prospective DBPCFC in 2%	Peanut	*	100%/14	57%	100%

Maloney JM et al[23]	234	57%	6.1	Prospective; clinical history, no challenges	Peanut	*	99%/13	60%	96%

* Not provided

In general, the magnitude of a food-specific IgE level cannot predict the severity of the clinical reaction [32]. However, there was one recent report demonstrating a significant correlation between the magnitude of specific IgE and severity of clinical reaction in egg allergic children. But there were a number of important exceptions to this association [33]. An inverse relationship was reported between the ratio of total peanut-specific IgE and challenge score to peanut allergy (r = -0.561) [34]. A study also found that the food specific to total IgE ratio was no more helpful than the specific IgE value in predicting the outcome of a food challenge [35].

The allergen-specific IgE antibody level can also aid in predicting the natural history of allergies to peanut [36], tree nuts [37], cow's milk [38] and hen's egg [39]. The rate of decline of hen egg and cow's milk-specific IgE level can help predict the resolution of the allergy [40].

### Food challenge

The DBPCFC has been long considered the "gold standard" for the diagnosis of food allergy and as a benchmark test from which to judge the diagnostic performance characteristics of the clinical history, skin test and IgE antibody serology. Open challenges may have false positive results ranging from 20.5-71% [41-43]. However, positive placebo reactions, that occur during the DBPCFC may be as high as 35% [44,45]. False-negative open challenges occur 1-3% of the time [2]. Some authors argue that performing several placebo and active oral provocations may be necessary to increase the specificity of DBPCFC to ~95% [3]. The same authors and others [46,47] point out that the general lack of standardized methods for the oral challenges is a primary limitation of the DBPCFC. Given a reported placebo reaction rate of 27% in adults undergoing oral drug challenge [48], oral food challenges in adults may have similar limitations. In summary, these limitations should be considered when estimating the overall diagnostic performance of SPT and specific IgE antibody testing.

### Cross-reactivity

IgE antibody (immunological) cross-reactivity between different foods or between food and aeroallergens such as trees and grasses occurs much more readily than clinically evident cross-reactivity. These immunological cross-reactions, which are seen with both skin testing and serological measures of IgE antibody, are generally reproducible and effectively inhibited by soluble allergen. However, they can often fail to translate into a clinical response following allergen exposure. Thus, a positive IgE antibody response that is associated with a cross-reaction may be considered a false positive result in relation to the subject's history of symptoms [49]. This further emphasizes the fact that the simple presence IgE antibody is necessary but not sufficient for clinical manifestation of allergic symptoms. Other factors such as the affinity, epitope specificity (extent of cross-reactivity), concentration and specific IgE to total IgE ratio all contribute to whether effector cells will degranulate following allergen exposure [50,51]. Patients, for example, who are sensitized to grass may have positive skin test to wheat, even though there is no evidence of clinical reactivity to the ingestion of wheat [52]. Sicherer provides an excellent review on the cross reactivity exhibited between foods [49].

Techniques to better understand the intricacies of cross reactivity remain one of the great challenges in the accurate laboratory diagnosis of food allergy. ISAC [28,29] is one IgE antibody assay that is specifically designed to aid the clinician in identifying the presence and quantifying the degree cross-reactive IgE antibody among the different food and pollen allergen groups that are known to share extensive homology. Bet-v 1 from Birch tree pollen, for instance, has structural homology in the PR10 family with allergenic proteins from alder tree pollen (Aln-g 1), hazelnut pollen (Cor-a 1), apple (Mal-d 1), peach (Pru-p 1), soybean (Gly-m 4), peanut (Ara-h 8), celery (Apr-g 1), carrot (Dau-c 1) and kiwi (Act-d 8). A primary sensitivity to Bet-v 1 may result in oral allergy symptoms after exposure to any of these other structurally similar (cross-reactive) allergenic molecules. The ISAC chip also can aid in identifying cross-reactivity among other allergen families such as the profilins (e.g., Bet-v 2-Birch, Ole-e 2-Olive, Hev-b 8-Latex, Phi-p 12-timothy grass), the lipid transfer proteins (e.g., Cor-a 9-hazelnut, Pru-p 3-peach, Art-v 3-mugwort and Par-j 2-Wall pellitory), the calcium binding proteins (e.g., Bet-v 4-birch, Phl-p 7-timothy grass), the tropomyosins (e.g, Pen-a 1-shrimp, Der-p 10-house dust mite, Bla-g 7-cockroach, Ani s 3-Anisakis), and the serum albumin family (e.g, Bos-d 6-bovine, Fel-d 2-cat, Can-f 3-dog, Equ-c 3-Horse and Gal-d 5-chicken). Knowledge of the extent of IgE cross-reactivity among these structurally similar proteins provides unique information to the allergist as support to the clinical history in diagnosis and management of the food allergic patient [53]. Combined with personal computer-based intelligent software algorithms that aid the practicing allergy specialist in digesting and interpreting the vast amount of IgE antibody data from the chip-based microarray assay, the issue of food cross-reactivity should become more manageable. One high profile serological issue involving PR-10/Bet v 1 homologue cross-reactivity is the recent supplementation of the hazelnut Phadia ImmunoCAP allergosorbent with recombinant hazelnut Cor a 1 that is known to cross-react with Birch Bet v 1 [54]. Following this supplementation, serum from birch pollen allergic individuals containing IgE anti-Bet v 1 produced high IgE anti-hazelnut levels in the ImmunoCAP due to cross-reactivity. The clinical significance of these levels has been questioned and some clinicians have returned to evaluating their patients for hazelnut sensitivity using Cor a 1 unsupplemented hazelnut allergosorbents.

### Epitope mapping in food allergy

Recent scientific advances have allowed for the identification and cloning of specific food epitopes [55]. The identification of specific IgE epitopes with immunoblot analyses may theoretically be used to better define the likelihood of clinical reactivity and/or natural history of food allergy than traditional allergen specific IgE measurements as described above in the section on "specific IgE testing" [55]. Special attention has been given to the quantitative detection of linear versus conformational food epitopes. One hypothesis is that conformational epitopes on food allergens may degrade in the GI tract, while linear epitopes retain their immunogenicity and allergenicity even in the enzyme rich, acidic gut environment [55]. Thus, children who have IgE antibodies specific for linear epitopes to alpha-s-1 and beta-casein, for instance, may be more likely to have persistent milk allergy [56]. Caseins comprise 80% of milk proteins and are composed of 4 protein fractions: α_s1_-, α_s2_-, β-, and κ-caseins. Whey proteins comprise the remainder of milk protein. The relative allergenicity of each cow's milk protein is unclear, although caseins seem to be the major allergen [57] Likewise, children with persistent hen egg allergy develop IgE antibodies against more sequential and conformational epitopes of ovomucoid, the dominant and most allergenic egg allergen, and ovalbumin [58]. However, epitope mapping of peanut allergens has not offered substantial clinical benefit over specific IgE measurements for the assessment of peanut allergy [59,60].

### Summary

Diagnosis of IgE-mediated food allergy has progressed over the last ten years. Threshold values for allergen-specific IgE have provided allergy specialists with a new diagnostic tool to define the need for a food challenge and allowed greater insight into the natural history of allergic reactions to selected foods. These IgE antibody threshold values should be carefully used, however, while taking into consideration the potential variability resulting from differences in the study populations and the methods used in provocation testing. Better definition of the IgE cross-reactivity among foods and between foods and pollens needs to be factored into the diagnostic process to more accurately predict clinical reactivity. Furthermore, use of recombinant and native purified allergenic molecules in the micro-array chip-based ISAC assay for specific IgE antibody should help clarify some common cross-reactivity seen among foods. Finally, exploration of food allergen epitope diversity and IgE avidity and specific activity (specific to total IgE ratio) may allow for improved diagnostic specificity.

## Competing interests

The authors declare that they have no competing interests.

## Authors' contributions

All authors read and approved the final manuscript.
